# Association Between Illness Severity Scores and Quantitatively Measured Brain Injury in Cardiac Arrest Survivors

**DOI:** 10.3390/jcm15093427

**Published:** 2026-04-30

**Authors:** Junho Lee, Jung Soo Park, Yeonho You, Jin Hong Min, So Young Jeon, Wonjoon Jeong, Changshin Kang

**Affiliations:** 1 Department of Emergency Medicine, Chungnam National University Hospital, 282, Munhwa-ro, Jung-gu, Daejeon 35015, Republic of Korea; junho4743@cnuh.co.kr (J.L.); cpcr@cnu.ac.kr (J.S.P.); yyo1003@naver.com (Y.Y.); laphir2006@naver.com (J.H.M.); chloe9899@cnuh.co.kr (S.Y.J.); 2 Department of Emergency Medicine, College of Medicine, Chungnam National University, Daejeon 35015, Republic of Korea

**Keywords:** out-of-hospital cardiac arrest, hypoxia-ischemia, brain, post-cardiac arrest syndrome, risk stratification, magnetic resonance imaging

## Abstract

**Introduction**: This study explored how illness severity scores correspond to hypoxic-ischemic brain injury (HIBI) after cardiac arrest. **Methods**: This study included cardiac arrest survivors with sufficient data to calculate the Pittsburgh Cardiac Arrest Category (PCAC) and revised post-cardiac arrest syndrome for therapeutic hypothermia (rCAST) scores who underwent brain magnetic resonance imaging and cerebrospinal fluid neuron–specific enolase (CSF-NSE) measurement within 6 h after return of spontaneous circulation. The primary outcome was the association of PCAC and rCAST with quantitative brain injury markers assessed using whole brain mean apparent diffusion coefficient (mean ADC), low ADC volume fractions (PV600, 650, and 700), and CSF-NSE. **Results**: In total, 81 patients were included. PCAC was not significantly associated with CSF-NSE, mean ADC, or PVs. The rCAST score was significantly associated with higher CSF-NSE, lower mean ADC, and higher PV700. The neurologic sub-score of PCAC was independently associated with all evaluated brain injury markers, whereas the systemic sub-score was not. Of the individual rCAST components, anoxic time was independently associated with CSF-NSE, whereas no other single component was associated with these markers. **Conclusions**: rCAST was significantly associated with degree of HIBI, whereas PCAC was not. The neurologic sub-score of PCAC showed independent associations with HIBI.

## 1. Introduction

In patients with post-cardiac arrest syndrome (PCAS), early prognostic assessment has been suggested to be useful in guiding the initial risk classification and optimizing the use of intensive care resources by distinguishing the illness severity of cardiac arrest [1]. Several early prognostication tools have been suggested to mitigate this risk [2,3,4,5,6,7,8,9]. Among them, the Pittsburgh Cardiac Arrest Category (PCAC) score is a practical one-to-four illness severity score that evaluates baseline neurological function and cardiopulmonary failure based on respiratory and hemodynamic support requirements [8]. Similarly, the Japanese-derived revised post-cardiac arrest syndrome for therapeutic hypothermia (rCAST) score was developed to provide early risk stratification as an illness severity score [9]. PCAC and rCAST scores have undergone external validation in independent cohorts and have demonstrated reliable prognostic performance [4,10,11,12,13,14]. Consequently, these early prognostication tools have been useful for illness severity stratification and guiding the selection of an optimal post-resuscitation care strategy in patients with PCAS [15,16,17].

A study that directly compared rCAST and PCAC scores within a single cohort reported superior performance of the rCAST score in predicting neurological outcomes compared with the PCAC score [1]. While their explanations are plausible, they did not directly address the pathobiological status of brain injury at the time of early prognostic assessment.

Therefore, the present study directly compared illness severity assessed by early prognostication tools with quantitatively measured brain injury within the same early post-resuscitation time window to understand how these illness severity scores correspond to hypoxic-ischemic brain injury (HIBI) due to cardiac arrest.

## 2. Patients and Methods

### 2.1. Study Design and Patients

This single-center, retrospective, observational, registry-based study was conducted at Chungnam National University Hospital (CNUH), a tertiary-care hospital in Daejeon, Republic of Korea. The institutional registry prospectively collected data on adult patients (age >18 years) who received post-resuscitation care after out-of-hospital cardiac arrest since January 2018. From this registry, patients enrolled between May 2018 and December 2024 were screened, and only those with sufficient data to calculate PCAC and rCAST scores who also underwent brain magnetic resonance imaging (MRI) and cerebrospinal fluid (CSF) neuron–specific enolase (NSE) measurement were included in this study. Patients were excluded if they had a pre-existing neurological disorder, first recording data obtained more than 6 h after return of spontaneous circulation (ROSC), or insufficient data quality for quantitative analysis of brain MRI findings.

This study was approved by the Institutional Review Board (IRB) of Chungnam National University Hospital (No. CNUH IRB 2024-06-046). The requirement for informed consent for this retrospective analysis of the registry data was waived by the IRB due to the observational nature of the study. However, as part of the prospectively collected registry protocol, informed consent for lumbar catheter placement was obtained from the legally authorized representative after a thorough medical evaluation confirmed that no contraindications to lumbar catheter placement were present (Table S1), owing to the characteristics of patients with cardiac arrest. Additionally, written informed consent was obtained from patients who achieved neurological recovery after post-resuscitation care. All procedures and protocols were conducted in accordance with the Declaration of Helsinki and the International Conference on Harmonization Good Clinical Practice guidelines.

### 2.2. Post-Resuscitation Care

Standardized post-resuscitation care was provided to all enrolled patients with a Glasgow Coma Scale motor (GCS-M) score < 6 after ROSC [18,19]. Temperature control was delivered using an external cooling device (Arctic Sun^®^ 5000; BD, Franklin Lakes, NJ, USA). The target temperature, selected by the attending physician (33 or 36 °C) according to the patient’s hemodynamic status and cardiac arrest characteristics, was maintained for 24 h, followed by controlled rewarming to 37 °C at 0.25 °C per hour. Esophageal or bladder probes were used for temperature monitoring. Sedation with midazolam and neuromuscular blockade with cisatracurium or rocuronium were routinely administered. Antiepileptic drugs were prescribed when seizures were diagnosed clinically or electrographically. Withdrawal of life-sustaining therapy is highly restricted in Korea.

### 2.3. Data Acquisition

#### 2.3.1. Baseline Characteristics

The following variables were extracted from the data registry: age, sex, Charlson comorbidity index, sequential organ failure assessment (SOFA) score during post-resuscitation care, witnessed collapse, bystander cardiopulmonary resuscitation (CPR), anoxic time (time from CPR to ROSC), first monitored rhythm, etiology of cardiac arrest, GCS score after ROSC, and time from ROSC to illness severity assessment, brain MRI, and CSF-NSE measurement.

#### 2.3.2. Stratification of Illness Severity

Among several early prognostication tools, PCAC and rCAST scores were used to stratify illness severity in this study population. Since 2020, PCAC and rCAST illness severity scores have been prospectively recorded in our data registry. For enrolled patients treated with post-resuscitation care before 2020, these scores were retrospectively calculated on the basis of the initial clinical and neurological data documented in the electronic medical records. All clinical parameters used to calculate these illness severity scores were obtained at the closest time to cardiac arrest, within 6 h after ROSC.

The PCAC score is derived from the full outline of unresponsiveness (FOUR) brainstem and motor sub-scores and SOFA cardiac and respiratory subscales [20,21], and it is documented as one of four illness severity categories. The formula for calculating the rCAST score is shown in Table S2. According to predefined rCAST score categories, patients were classified as having low (≤5.5), moderate (6.0–14.0), or high (≥14.5) severity [9].

#### 2.3.3. Quantitative Assessment of Brain Injury

The pathobiological degree of HIBI was quantitatively assessed using brain MRI-based apparent diffusion coefficient (ADC) analysis and CSF-derived NSE measurement within the same early post-resuscitation time window. The CSF sample was obtained through lumbar catheters (HermeticTM lumbar accessory kit; Integra Neurosciences, Plainsboro, NJ, USA). NSE was measured using an electro-chemiluminescence immunoassay kit (COBAS^®^ e801; Roche Diagnostics, Basel, Switzerland), and its measurement ranged from 0.1–300 ng/mL. As this procedure is not part of routine post-resuscitation care, patients were closely monitored for potential complications.

At our institution, the standard protocol for post-resuscitation care recommends obtaining brain MRI before post-resuscitation care. MRI was performed using a 3 T scanner (Intera Achieva; Philips Healthcare, Best, The Netherlands), which included diffusion-weighted image (DWI), ADC maps, and T2-weighted imaging. DWI with b-values of 0, 1000, and 3000 is conventionally performed in the axial plane using three orthogonal directions of the diffusion-sensitizing gradients combined into isotropic images. For voxel-based quantitative analysis of ADC, FSL software (Release 5.0 © 2012; The University of Oxford, Oxford, UK) was used to semi-automatically remove the cranium, optic structures, and extracranial soft tissues. Images were retrieved in Digital Imaging and Communication in Medicine format from picture archiving and communication system servers and were converted to the Neuroimaging Informatics Technology Initiative format using MRIcron software (http://www.nitrc.org/ projects/mricron, accessed on 1 January 2025). Then, we calculated the average ADC values in the entire brain volume (mean ADC) and percentage of voxels below different ADC thresholds (PV). To reduce errors caused by artifacts, noise, and fluid content, voxels with ADC values > 1200 × 10^−6^ mm^2^/s were extracted from the analysis [22,23]. The ADC thresholds ranged from 200–1200 × 10^−6^ mm^2^/s with a step of 50, and the PV values below the threshold (PV[x]) were calculated for each ADC threshold up to 1150 × 10^−6^ mm^2^/s [23]. To calculate PV(x), the total sum of the voxels from 200 to the specified interval was calculated, and then the value was divided by the total sum of the voxels within the range of 200–1200 [23]. The equation of PV(x) is as follows:

$$PV(x),\%=\frac{Ʃ(Voxels\;with\;ADC\;value\;between\;200\;and\;x)}{Ʃ(Voxels\;with\;ADC\;value\;between\;200\;and\;1200)}\times 100$$

PV600, PV650, and PV700 have been reported as indicating significant prognostic performance in cardiac arrest survivors undergoing post-resuscitation care [24,25]; therefore, these indices were included as quantitative imaging markers of brain injury.

### 2.4. Outcomes

The primary outcome was the association between illness severity assessed by PCAC and rCAST scores and MRI-derived and CSF-derived quantitative measures of brain injury. The secondary outcome was to determine which sub-score or component of the PCAC score (the neurologic [P-FOUR] and cardiovascular [P-SOFA] components) and rCAST score (initial rhythm, anoxic time, arterial pH, lactate level, and GCS-M score) independently contributed to the association between the overall illness severity scores and quantitative brain injury markers. The neurological outcomes were prospectively assessed for clinical follow-up using cerebral performance categories (CPCs), which classify patients into CPC 1 (good performance), CPC 2 (moderate disability), CPC 3 (severe disability), CPC 4 (vegetative state), and CPC 5 (brain death or death). The results were dichotomized as either good (CPC 1–2) or poor (CPC 3–5). An investigator unaffiliated with this study assessed CPC at 6 months by semi-structured interviews with patients or their relatives.

### 2.5. Statistical Analysis

Categorical variables are reported as frequency with percentage and continuous variables as median with interquartile range (IQR) because the distributions were non-normal. Categorical variables were compared using chi-square tests. The Kruskal–Wallis test with Bonferroni-adjusted post-hoc comparisons was applied across severity groups, separately for PCAC (categories 2–4) and rCAST (low, moderate, and high). Because ADC-derived metrics and low-ADC volume measures are strictly positive and demonstrated right-skewed distributions, generalized linear models (GLMs) with a gamma distribution and log link function were used to evaluate the associations between illness severity scores and quantitative brain injury markers. Separate multivariable models were constructed for each dependent variable, including CSF-NSE, mean ADC, PV600, PV650, and PV700. All analyses were performed using a complete-case approach without data imputation. Model results are reported as regression coefficient on the log scale with corresponding 95% confidence interval (CI). Exponentiated coefficients (exp [β]) were interpreted as multiplicative effects on the dependent variable. The primary predictors were PCAC and rCAST scores, each analyzed in separate models as categorical variables. To account for potential confounding, models were adjusted for clinically relevant baseline variables, including age, sex, witnessed arrest, bystander cardiopulmonary resuscitation, and initial rhythm. Variables that were components of the respective illness severity scores were not included in the adjusted models to avoid multicollinearity. Model performance was assessed using the Cox–Snell pseudo-R^2^ value, deviance, and Pearson chi-square statistic. A two-sided *p*-value < 0.05 was considered statistically significant, and statistical analyses were performed using Python (version 3.11) with the pandas, NumPy, and statsmodels (version 0.14.x) libraries.

## 3. Results

### 3.1. Baseline Patient Characteristics

**Figure 1:**
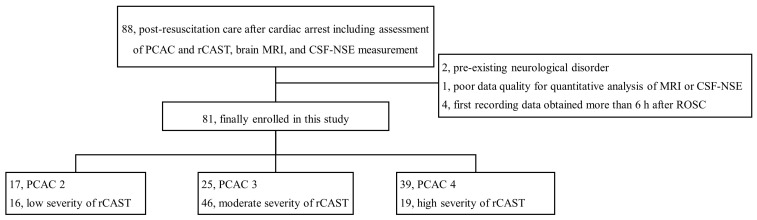
Flow diagram of the included study population.

In total, 81 patients were included in the analysis (Figure 1). Baseline demographics were comparable across all subgroups (Table 1). Regarding cardiac arrest characteristics, significant differences were observed according to PCAC stages. Compared with patients in PCAC 2 and 3, those in PCAC 4 demonstrated a significantly lower prevalence of shockable rhythm (versus [vs.] PCAC 2, 12.8% vs. 56.2%, *p* = 0.008; vs. PCAC 3, 12.8% vs. 44.0%, *p* = 0.04; Table 1) and a longer median anoxic time (vs. PCAC 2, 23.0 min vs. 9.0 min, *p* = 0.01; vs. PCAC 3, 23.0 min vs. 15.0 min, *p* = 0.03; Table 1). Similarly, the high severity of rCAST was characterized by a significantly lower rate of witnessed arrests (vs. low severity, 21.1% vs. 93.8%, *p* < 0.001; vs. moderate severity, 21.1% vs. 67.4%, *p* = 0.005; Table 1) than low and moderate severities and a prolonged anoxic time compared with low severity (25.0 min vs. 10.5 min, *p* = 0.002; Table 1).

Neurological outcomes significantly differed across subgroups. Poor neurological outcome was most frequent in PCAC 4 (82.1%) and high severity (84.2%) (both, *p* < 0.001; Table 1), with post-hoc analysis identifying these as the primary poor neurological outcome groups. Although omnibus test results indicated overall variability in cardiac death rates (between PCAC groups, *p* = 0.009; between rCAST groups, *p* = 0.02), strict post-hoc analysis with Bonferroni correction revealed no significant pairwise differences between specific subgroups. No significant differences were observed in neurological death rates (all, *p* > 0.05).

Figure 1 presents a flow diagram showing patient selection from the registry of out-of-hospital cardiac arrest patients receiving post-resuscitation care. Of 88 patients who underwent PCAC and rCAST assessments, brain MRI, and CSF-NSE measurement, 7 were excluded (pre-existing neurological disorder, n = 2; insufficient data quality for quantitative MRI or CSF-NSE analysis, n = 1; first recorded data obtained >6 h after ROSC, n = 4). A total of 81 patients were included in the final analysis and categorized according to PCAC (PCAC 2, n = 17; PCAC 3, n = 25; PCAC 4, n = 39) and rCAST severity groups (low, n = 16; moderate, n = 46; severe, n = 19).

### 3.2. Association of Illness Severity with Quantitative Measures of Brain Injury

Associations between cardiac arrest illness severity assessed by PCAC and rCAST scores and CSF-derived and MRI-derived quantitative markers of brain injury are shown in Table 2. PCAC was not significantly associated with CSF-NSE (β = 24.0 [95% CI: −3.1 to 51.2], *p* = 0.08), mean ADC (β = −3.6 [95% CI: −14.1 to 6.8], *p* = 0.49), or PV700 (β = 1.8 [95% CI: −1.0 to 4.5], *p* = 0.21). Borderline associations were observed for PV600 (β = 1.5 [95% CI: −0.03 to 3.1], *p* = 0.05) and PV650 (β = 1.9 [95% CI: −0.2 to 4.1], *p* = 0.08). By contrast, the rCAST score was significantly associated with several markers of brain injury, including higher CSF-NSE levels (β = 34.3 [95% CI: 1.8 to 66.8], *p* = 0.04, pseudo-R^2^ = 0.14), lower mean ADC (β = −15.4 [95% CI: −27.9 to −2.9], *p* = 0.02, pseudo-R^2^ = 0.11), and higher PV700 (β = 3.5 [95% CI: 0.3 to 6.8], *p* = 0.04, pseudo-R^2^ = 0.11).

### 3.3. Association of Sub-Scores in Early Prognostication Tools with Quantitative Measures of Brain Injury

Additional analyzes were performed to determine which sub-scores in each early prognostication tools accounted for these associations (Table 3 and Table 4). In the PCAC component model, P-FOUR was independently associated with all evaluated markers, including CSF-NSE (β = −20.2 [95% CI: −33.6 to −6.8], *p* = 0.003, pseudo-R^2^ value = 0.11; Table 3), mean ADC (β = 5.9 [95% CI: 0.7 to 11.1], *p* = 0.03, pseudo-R^2^ = 0.06; Table 3), PV600 (β = −0.8 [95% CI −1.6 to −0.1], *p* = 0.04, pseudo-R^2^ = 0.07; Table 3), PV650 (β = −1.2 [95% CI: −2.2 to −0.1], *p* = 0.03, pseudo-R^2^ = 0.06; Table 3), and PV700 (β = −1.5 [95% CI: −2.9 to −0.1], *p* = 0.04, pseudo-R^2^ = 0.05; Table 3), whereas P-SOFA was not associated with any marker (all, *p* > 0.05; Table 3). In the rCAST component model, anoxic time was independently associated with CSF-NSE (β = 36.6 [95% CI: 13.7 to 59.6], *p* = 0.002, pseudo-R^2^ = 0.23; Table 4). No individual rCAST sub-scores showed a significant association with mean ADC or PV metrics, although the GCS-M score showed borderline associations across MRI-derived variables.

## 4. Discussion

Here, the main finding was that the rCAST score showed significant associations with the degree of HIBI after cardiac arrest. By contrast, the PCAC score was not significantly associated with these MRI-derived or CSF-derived measures. Previous studies have mainly examined these early prognostication tools in relation to clinical outcomes [1,10,11], whereas this study evaluated their correspondence with the pathobiological burden of HIBI using voxel-based MRI analysis and CSF-NSE measurement. A major strength of this study is the direct comparison of early illness severity scores with objective MRI-derived and CSF-derived markers of brain injury obtained in near temporal proximity, allowing these prognostication tools to be interpreted against the pathobiological degree of HIBI at nearly the same time point. These findings may provide further insight into how early prognostication tools relate to the pathobiological severity of brain injury in the early post-resuscitation period after cardiac arrest while also highlighting considerations for their appropriate interpretation and clinical use.

The PCAC score showed no meaningful association with the quantitative degree of brain injury, whereas the rCAST score was significantly associated with quantitative brain injury markers. This discrepancy may reflect differences in the design of the two tools. PCAC was developed to reflect neurologic injury and cardiopulmonary dysfunction [14], whereas rCAST was originally developed to estimate neurologic outcome rather than mortality [9,13]. However, this should not be interpreted as indicating that the PCAC score is unrelated to HIBI after cardiac arrest. Although the PCAC score incorporates cardiopulmonary dysfunction in addition to neurologic injury, greater weight is placed on the neurologic component [14]. Consistent with this, its neurologic sub-score, P-FOUR, was independently associated with MRI-derived and CSF-derived markers of brain injury, and its explanatory power for CSF-NSE was comparable to that of rCAST. These findings may provide supportive evidence for the original rationale underlying the development of each tool. Many clinicians use early prognostication tools to guide initial risk classification and optimize the allocation of intensive care resources by distinguishing the illness severity of cardiac arrest. However, substantial concerns remain regarding the risk of pessimistic prognostication due to an inaccurately negative prognosis [19,26]. Our results suggest that when prognostic assessment is focused specifically on the degree of brain injury rather than overall systemic severity, greater emphasis may need to be placed on tools that more directly correspond to the pathobiological burden of HIBI itself. Although the full FOUR score [20] could not be analyzed owing to data limitation in our registry, P-FOUR showed significant associations with quantitative brain injury markers, with explanatory power comparable to that of rCAST. Additionally, previous studies have reported the superior prognostic performance of the full FOUR score compared with PCAC and rCAST [1]. Therefore, we suggest that neurologically focused measures may provide a more reliable basis for neurologically focused prognostic assessment than composite scores influenced by extracranial organ dysfunction and may help provide accurate guidance for initial risk classification and optimizing the use of medical resource by distinguishing the illness severity of cardiac arrest according to the degree of HIBI due to cardiac arrest.

Among the individual rCAST components, only anoxic time was independently associated with CSF-NSE levels, with robust explanatory power, whereas the GCS-M score was not significantly associated with any quantitative brain injury marker. We further analyzed anoxic time as a continuous variable based on actual low-flow time, and in both analyses, continuous low-flow time remained independently associated with quantitative brain injury markers, whereas the GCS-M score still showed no significant association. Although the GCS-M score had the greatest weighting among the individual rCAST components in the original score derivation for neurologic outcome prediction [2,9], it did not show a corresponding association, in which the outcome was the pathobiological burden of brain injury itself, not the clinical outcome. Similarly, previous studies have reported an independent association between anoxic time and clinical outcomes after cardiac arrest [27,28]. However, given the limited sample size of our cohort, we cannot make definitive conclusions regarding the relationship between individual rCAST components and the degree of brain injury. Therefore, future studies should clarify which individual rCAST components best reflect the pathobiological burden of HIBI.

Early prognostication after cardiac arrest is of critical importance for guiding clinical decision-making, optimizing the allocation of intensive care resources, and facilitating communication with patients’ families. However, there remains a substantial risk of inaccurate or overly pessimistic prognostic assessment, particularly when tools are applied without sufficient consideration of their underlying biological relevance. In this context, prognostic tools that more closely reflect the structural and functional extent of HIBI may provide more reliable guidance in the early post-resuscitation period. Our findings support the notion that integrating objective measures of brain injury, such as quantitative MRI-derived metrics and CSF biomarkers, with clinically applicable prognostic tools may enhance the accuracy and clinical utility of early risk stratification.

Our study has some limitations. As a single-center, retrospective, observational study, the findings may not be fully generalizable to other settings with different patient populations, pre-hospital systems, or post-resuscitation care protocols. The sample size was also small. Additionally, selection bias cannot be excluded. Although PCAC and rCAST scores were prospectively documented in the registry after 2020, some scores in earlier patients were retrospectively calculated from electronic medical records, which may have introduced measurement bias. Further, the timing of illness severity assessment and quantitative brain injury measurement did not perfectly coincide, which may have introduced temporal variability despite all measurements being obtained within the same ultra-early post-resuscitation window. Finally, although all data were obtained within 6 h after ROSC, biological markers of brain injury may still have been evolving during this ultra-early period.

## 5. Conclusions

In the early post-resuscitation care period, the rCAST score was significantly associated with quantitative MRI-derived and CSF-derived brain injury markers, whereas the PCAC score was not. However, the neurologic sub-score of PCAC (P-FOUR) showed independent associations with these markers. These findings suggest that neurologically focused measures may more closely reflect the pathobiological burden of HIBI and may have potential clinical utility in early prognostic assessment after cardiac arrest. Further studies are warranted to confirm these findings.

## Figures and Tables

**Table 1:** Baseline demographics and characteristics.

Variable	Total Cohort, n = 81	PCAC			rCAST		
		PCAC 2, n = 17	PCAC 3, n = 25	PCAC 4, n = 39	Low, n = 16	Moderate, n = 46	High, n = 19
Age, y	56 (40–66)	51 (35–66)	54 (41–63)	57 (42–65)	58 (40–68)	57 (42–64)	45 (40–62)
Sex, male	63 (77.8)	14 (82.4)	19 (76.0)	30 (76.9)	15 (93.8)	34 (73.9)	14 (73.7)
Charlson Comorbidity Index	1 (0–4)	1 (0–4)	1 (0–3)	2 (1–5)	2 (1–5)	2 (1–5)	1 (0–2)
Cardiac arrest characteristic							
Witnessed	50 (61.7)	14 (82.4)	18 (72.0)	18 (46.2)	15 (93.8)	31 (67.4)	4 (21.1) ***^,††^
Bystander CPR	55 (67.9)	12 (70.6)	20 (80.0)	23 (59.0)	12 (75.0)	32 (69.6)	11 (57.9)
Shockable rhythm	25 (31.2)	9 (56.2)	11 (44.0)	5 (12.8) **^,†^	10 (62.5)	10 (22.2) *	5 (26.3)
Cardiac etiology	29 (35.8)	9 (52.9)	10 (40.0)	10 (25.6)	10 (62.5)	13 (28.3)	6 (31.6)
Anoxic time, min	19.0 (9.0–27.0)	9.0 (5.0–20.0)	15.0 (8.0–22.0)	23.0 (15.0–31.5) *^,†^	10.5 (7.8–15.8)	17.5 (9.0–25.0)	25.0 (19.5–31.0) **
Time from ROSC							
to MRI, h	4.2 (3.1–6.0)	3.57 (3.22–4.85)	4.08 (3.23–5.75)	4.5 (4.0–6.1)	4.37 (3.34–7.34)	4.5 (3.1–6.1)	4.0 (3.1–4.2)
to measure CSF-NSE, h	4.4 (3.5–5.8)	3.9 (3.0–4.7)	4.2 (2.7–6.1)	4.6 (4.0–5.9)	4.6 (3.7–8.0)	4.7 (4.0–6.0)	4.1 (3.2–4.6)
to induction, h ^a^	6.0 (4.7–8.2)	7.5 (5.8–9.7)	6.1 (4.9–8.3)	5.3 (4.4–7.2)	6.9 (5.8–9.4)	6.0 (4.5–8.4)	5.2 (4.5–6.3)
to assess illness severity, min	60 (17–146)	85 (34–206)	29 (14–159)	61 (21–125)	131 (23–212)	81 (17–145)	21 (10–53)
Neurological outcome							
Good (CPC 1 and 2)	38 (46.9)	15 (88.2)	16 (64.0)	7 (17.9) ***^,††^	15 (93.8)	20 (43.5) **	3 (15.8) ***
Poor (CPC 3 to 5)	43 (53.1)	2 (11.8)	9 (36.0)	32 (82.1)	1 (6.2)	26 (56.5)	16 (84.2)
Cardiac death ^b^	21 (25.9)	1 (5.9)	4 (16.0)	16 (41.0)	1 (6.2)	11 (23.9)	9 (47.4)
Neurologic death	11 (13.6)	0	3 (12.0)	8 (20.5)	0 (0.0)	7 (15.2)	4 (21.1)

Data are presented as n (%) or median (interquartile range). ^a^ Time to reach targeted temperature (33 or 36 °C) from ROSC. ^b^ Although omnibus tests indicated significance, strict post-hoc analysis revealed no significant pairwise differences between specific subgroups. * Statistical significance at <0.05 compared with PCAC 2 or low severity of rCAST in post-hoc Bonferroni analysis. ** Statistical significance at <0.01 compared with PCAC 2 or low severity of rCAST in post-hoc Bonferroni analysis. *** Statistical significance at <0.001 compared with PCAC 2 or low severity of rCAST in post-hoc Bonferroni analysis. ^†^ Statistical significance at <0.05 compared with PCAC 3 or moderate severity of rCAST in post-hoc Bonferroni analysis. ^††^ Statistical significance at <0.01 compared with PCAC 3 or moderate severity of rCAST in post-hoc Bonferroni analysis. **Abbreviations**: PCAC, Pittsburgh Cardiac Arrest Category; rCAST, revised post-cardiac arrest syndrome for therapeutic hypothermia; CPR, cardiopulmonary resuscitation; ROSC, return of spontaneous circulation; MRI, magnetic resonance image; CSF-NSE, cerebrospinal fluid–neuron-specific enolase; CPC, cerebral performance category. Data are presented as n (%) or median (interquartile range).

**Table 2:** GLM analyses evaluating the associations of PCAC and rCAST scores with quantitative measures of brain injury.

Predictor	Outcome	β Coefficient	95% CI	*p*-Value	Pseudo-R^2^
PCAC ^a^	CSF-NSE	24.0	−3.1 to 51.2	0.08	.
	mean ADC	−3.7	−14.1 to 6.8	0.49	.
	PV600	1.5	0.0 to 3.1	0.05	.
	PV650	1.9	−0.2 to 4.1	0.08	.
	PV700	1.8	−0.1 to 4.5	0.21	.
rCAST ^a^	CSF-NSE	34.3	1.8 to 66.8	0.04	0.14
	mean ADC	−15.4	−27.9 to −2.9	0.02	0.11
	PV600	1.1	−0.8 to 2.9	0.27	.
	PV650	2.0	−0.5 to 4.6	0.12	.
	PV700	3.5	0.3 to 6.8	0.04	0.11

^a^ These predictors were categorized into three categories (e.g., PCAC, 2 to 4; rCAST, low to high). **Abbreviations**: GLM, generalized linear models; PCAC, Pittsburgh Cardiac Arrest Category; rCAST, revised post-cardiac arrest syndrome for therapeutic hypothermia; CSF-NSE, cerebrospinal fluid neuron–specific enolase; ADC, apparent diffusion coefficient; PV600, percentage of brain volume with ADC values < 600 × 10^−6^ mm^2^/s; PV650, percentage of brain volume with ADC values < 650 × 10^−6^ mm^2^/s; PV700, percentage of brain volume with ADC values < 700 × 10^−6^ mm^2^/s.

**Table 3:** GLM analyses evaluating the associations of sub-scores in PCAC (P-FOUR and P-SOFA scores) with quantitative measures of brain injury.

Predictor	Outcome	β Coefficient	95% CI	*p*-Value	Pseudo-R^2^
P-FOUR score ^a^	CSF-NSE	−20.2	−33.6 to −6.8	0.003	0.11
	mean ADC	5.9	0.7 to 11.1	0.03	0.06
	PV600	−0.8	−1.6 to −0.1	0.04	0.07
	PV650	−1.2	−2.2 to −0.1	0.03	0.06
	PV700	−1.8	−2.9 to −0.1	0.04	0.05
P-SOFA score ^b^	CSF-NSE	1.1	−6.2 to 8.4	0.76	
	mean ADC	1.7	−1.2 to 4.5	0.25	
	PV600	0.2	−0.2 to 0.6	0.40	
	PV650	0.1	−0.5 to 0.7	0.68	
	PV700	−0.1	−0.9 to 0.6	0.76	

^a^ The neurologic sub-score of PCAC (P-FOUR) was categorized into three categories (8, 4–7, and <4). ^b^ The cardiopulmonary sub-score of PCAC (P-SOFA) was categorized into two categories (<4 and ≥4). **Abbreviations**: GLM, generalized linear models; PCAC, Pittsburgh Cardiac Arrest Category; FOUR, full outline of unresponsiveness; SOFA, sequential organ failure assessment; CSF-NSE, cerebrospinal fluid neuron-specific enolase; ADC, apparent diffusion coefficient; PV600, percentage of brain volume with ADC values < 600 × 10^−6^ mm^2^/s; PV650, percentage of brain volume with ADC values < 650 × 10^−6^ mm^2^/s; PV700, percentage of brain volume with ADC values < 700 × 10^−6^ mm^2^/s.

**Table 4:** GLM analyses evaluating the associations of sub-scores in rCAST (pH, lactate, GCS-M score, initial rhythm, and anoxic time) with quantitative measures of brain injury.

Predictor	Outcome	β Coefficient	95% CI	*p*-Value	Pseudo-R^2^
pH ^a^	CSF-NSE	8.8	−17.8 to 35.3	0.52	
	mean ADC	−6.6	−17.3 to 4.2	0.23	
	PV600	0.1	−1.5 to 1.7	0.92	
	PV650	0.7	−1.5 to 2.9	0.55	
	PV700	1.6	−1.2 to 4.5	0.26	
lactate ^a^	CSF-NSE	−10.3	−40.5 to 19.8	0.50	
	mean ADC	6.5	−5.7 to 18.7	0.30	
	PV600	−0.8	−2.6 to 1.0	0.38	
	PV650	−1.3	−3.8 to 1.2	0.31	
	PV700	−1.79	−5.0 to 1.4	0.27	
GCS-M ^a^	CSF-NSE	44.5	−9.9 to 99.0	0.11	
	mean ADC	−19.1	−41.1 to 3.0	0.09	
	PV600	2.8	−0.5 to 6.0	0.10	
	PV650	3.8	−0.7 to 8.4	0.10	
	PV700	4.8	−1.0 to 10.6	0.10	
initial rhythm ^a^	CSF-NSE	40.5	−1.6 to 82.7	0.06	
	mean ADC	−6.4	−23.4 to 10.7	0.46	
	PV600	1.4	−1.1 to 3.9	0.28	
	PV650	1.4	−2.1 to 4.9	0.43	
	PV700	1.3	−3.2 to 5.8	0.56	
anoxic time ^a^	CSF-NSE	36.6	13.7 to 59.6	0.002	0.23
	mean ADC	−5.9	−15.2 to 3.4	0.21	
	PV600	1.1	−0.3 to 2.5	0.13	
	PV650	1.5	−0.4 to 3.4	0.12	
	PV700	1.9	−0.5 to 4.4	0.12	

^a^ These individual components of the rCAST score were transformed into categorical variables with two, three, or four levels based on the predefined rCAST formula. **Abbreviations**: GLM, generalized linear models; rCAST, revised post-cardiac arrest syndrome for therapeutic hypothermia; GCS-M, Glasgow Coma Scale motor; CSF-NSE, cerebrospinal fluid neuron–specific enolase; ADC, apparent diffusion coefficient; PV600, percentage of brain volume with ADC values < 600 × 10^−6^ mm^2^/s; PV650, percentage of brain volume with ADC values < 650 × 10^−6^ mm^2^/s; PV700, percentage of brain volume with ADC values < 700 × 10^−6^ mm^2^/s.

## Data Availability

The data presented here are available on request from the corresponding author. The data are not publicly available because of ethical concerns.

## Supplementary Materials

The following supporting information can be downloaded at: https://www.mdpi.com/article/10.3390/jcm15093427/s1, Table S1: Contraindications for lumbar catheter placement; Table S2: Calculation of rCAST.

## Abbreviations

The following abbreviations are used in this manuscript:

PCAS	post-cardiac arrest syndrome
PCAC	Pittsburgh Cardiac Arrest Category
rCAST	revised post-cardiac arrest syndrome for therapeutic hypothermia
HIBI	hypoxic-ischemic brain injury
MRI	magnetic resonance imaging
CSF	cerebrospinal fluid
NSE	neuron-specific enolase
ROSC	return of spontaneous circulation
GCS-M	Glasgow Coma Scale motor
SOFA	sequential organ failure assessment
CPR	cardiopulmonary resuscitation
FOUR	full outline of unresponsiveness
ADC	apparent diffusion coefficient
DWI	diffusion-weighted image
PV	percentage of voxels
CPC	cerebral performance categories
IQR	interquartile range
GLM	generalized linear models
CI	confidence interval.
