# Supplementary material for: Association Between Illness Severity Scores and Quantitatively Measured Brain Injury in Cardiac Arrest Survivors
Source: J Clin Med. 2026 Apr 30;15(9):3427. doi: 10.3390/jcm15093427 (PMC13164181; doi:10.3390/jcm15093427)
Supplement: Supplementary file 1 [file jcm-15-03427-s001.zip › jcm-4272421-supplementary.pdf]

Supplemental Online Content

Association between Illness Severity Scores and Quantitatively Measured Brain Injury in Cardiac Arrest Survivors

Table S1. Contraindications for lumbar catheter placement

Table S2. Calculation of rCAST.

Table S1. Contraindications for lumbar catheter placement

**Contraindications**
Patient with uncontrolled diabetes
Effacement of the basilar cisterns (suprasellar, ambient, chiasmatic, and quadrigeminal)
Patients with coagulopathy
Thrombocytopenia (platelet count < 100,000)
Patients with a documented history of cirrhosis
Patients on low-molecular-weight heparin
Patients on clopidogrel bisulphate (Plavix) or other chronic platelet inhibitors
History of posterior fusion hardware that would interfere with catheter placement
Local skin infections or eruption over the puncture site
Sign of systemic infection or sepsis
Lumbar puncture within 6 h

Table S2. Calculation of rCAST.

A. Categorization of each variables

Score	0	1	2	3
initial rhythm	Shockable	Non-shockable		
witness/until ROSC time	< 20 min	20 min ≤	No witness	
pH	≥ 7.31	7.30–7.16	7.15–7.01	≤ 7.00
lactate	≤ 5.0	5.1–10.0	10.1–14.0	≥ 14.1
GCS-M	≥ 2	1		

B. Formula for calculation of rCAST

1.0 × (initial rhythm score)

+ 2.0 × (witness/until ROSC time score)

+ 2.5 × (pH score)

+ 0.5 × (lactate score)

+ 4.5 × (GCS-M score)

= rCAST score points

Abbreviations: ROSC, return of spontaneous circulation; GCS-M, motor scale of the Glasgow Coma Scale.
